# *“Helping myself empowered me to help young people better*”*:* A stepped care model, with non-specialist workers (NSWs) addressing mental health of young people in urban vulnerable communities across the Mumbai metropolitan region in India

**DOI:** 10.1017/gmh.2024.96

**Published:** 2024-10-24

**Authors:** Rama Shyam, Arati Mitra, Seema Sharma, Vinita Ajgaonkar, Anu Balasubramanyam, Anuja Jayaraman, Neeta Karandikar, Nikhat Shaikh, Sheetal Rajan, Tanushree Das, Tanya Raj

**Affiliations:** 1 Society for Nutrition, Education and Health Action (SNEHA), Mumbai, India; 2 Azim Premji University, Bangalore, India

**Keywords:** adolescent health, stigma, non-specialist worker, social determinants, urban community-based mental health

## Abstract

Research on adolescent mental health in low and middle-income countries cites the paucity of human resources and emphasises non-specialist worker (NSW)-led counselling intervention within school and health-system platforms. This pilot study aimed to evaluate the feasibility and acceptability of a transdiagnostic stepped care model, for delivering preventive psychological treatment to adolescents through NSWs in urban vulnerable community settings. Conducted in three such settlements in Mumbai and Thane districts of Maharashtra in India, this mixed-methods study engaged 500 young people, their parents and 52 NSWs.

Quantitative data, obtained through monitoring indicators, fidelity checklists and the Strengths and Difficulties Questionnaire (SDQ), revealed key stressors for adolescents, including poverty, structural inequity, cultural conformity pressures, academic anxieties and communication gap within families. Post-intervention, adolescents exhibited an enhanced capacity for positive emotions and agency. The qualitative component, incorporating observations, focus group discussions (FGDs) and in-depth interviews (IDIs) with various stakeholders, highlighted reduced stigma around mental health, yet identified barriers like time commitment, lack of incentivisation for NSWs, lack of privacy in densely populated communities and societal stigma.

This implementation research underscores that adolescent mental health stressors often originate from social determinants, exacerbated by insufficient awareness and stigma. Such stepped care models offer a pathway for communities to establish enduring support networks.

## Graphical abstract

**Figure:**
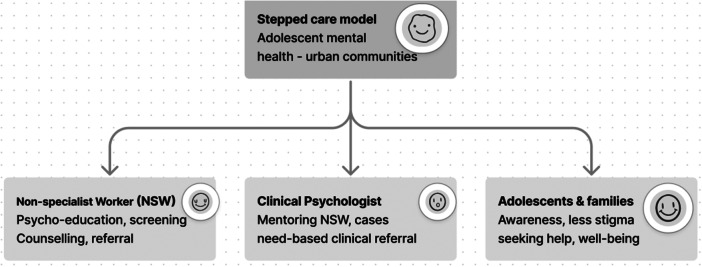

## Impact statement

The *Rashtriya Kishor Swasthya Karyakram* (RKSK) is India’s only national adolescent health policy, focusing on holistic adolescent outcomes. Through a network of Adolescent Friendly Health Clinics (AFHCs) located in the vicinity of 1 per 5,000 population, it proposed to address adolescents’ mental health issues – besides physical and sexual and reproductive health services – through first-level counselling and referral. In practice, these clinics are scarce and mainly focus on sexual and reproductive health. The second initiative under this policy is the recruitment and training of peer educators, who, while engaging in promotion activities, do not currently support screening, treatment or referrals for mental health problems.

The SAMWAAD (dialogue/conversation in Hindi and an acronym for Stepped cAre Model to Work on All that Ails the minD) study by SNEHA was aimed at building a transdiagnostic stepped care model to deliver psychological treatment for adolescents through non-specialist workers (NSWs) in urban vulnerable community settings. This implementation research study tested the acceptability and feasibility of running a community-based mental health intervention, the findings from which can be layered into the peer-educator and AFHC components under the national adolescent health policy.

Findings indicate that experiential learning and mentoring support can enable NSWs in their own experiences of mental health by breaking the stigma barriers. Mental health is affected by social determinants such as structural deprivations and factors beyond just the biomedical aspects. The ability of NSWs to address mental health concerns, along with social determinants, has shown an improvement in adolescents’ ability to feel positive and exercise their own agency. The research and intervention processes have been able to raise awareness on mental health among parents, improving communication with their children and supporting them through adolescence.

## Social media summary

Non-specialist workers providing psychoeducation, counselling and referral support to young people and their families can address mental health concerns effectively.

## Introduction

Adolescence is a formative phase of life during which patterns of growth, development and behaviour lay a foundation for health in later life (Widick et al., 1978). Addressing adolescent mental health is crucial to breaking the intergenerational cycle of poverty (Lund et al., 2023) and ensuring a life of well-being. An urgent priority in a country like India where every fifth person is an adolescent (Chandramouli and General, 2011), depression, anxiety and behavioural disorders are among the leading causes of illness and disability; suicide is among the leading causes of mortality among young people in India (Gupta and Basera, 2021). Estimates suggest a 7.3% psychiatric morbidity and treatment gaps over 75% among 13–17-year-olds (Gaiha et al., 2020), necessitating a task-sharing approach.

India is among the few low- and middle-income countries with progressive legislation and a policy on mental health (Roy et al., 2019). However, for adolescents in school and out of school, mental health services are almost non-existent (Gulliford et al. 2002), with stigma, non-participation of young people in policy development and paucity of qualified mental health workers as some of the key constraints (Roy et al., 2019; Mehra et al., 2022). India’s national adolescent health programme is aimed at addressing mental health issues of young people at the community level through a network of Adolescent Friendly Health Clinics (AFHCs). In principle, AFHCs should provide informal counselling for mental health problems and refer adolescents with severe problems to tertiary care (Ministry of Health and Family Welfare, 2014). In practice, AFHCs are scarce, mainly focused on sexual and reproductive health and seldom used by adolescents (Barua et al., 2020). Peer educators (Ministry of Health and Family Welfare, 2014), a part of the AFHC mandate, engage in promotion activities but do not currently support screening, treatment or referral for mental health problems (Barua et al., 2020).

In India, MANAS was a successful intervention (Patel et al., 2010) and is being adapted for adolescents in Nepal. The “Friendship Bench” problem-solving therapy led to strong reductions in anxiety and depression in adults in Zimbabwe (Chibanda et al., 2015). A transdiagnostic problem-solving intervention developed within the Premium for Adolescents (PRIDE) programme was piloted with 13–20-year-old students in New Delhi secondary schools and found to be feasible and acceptable (Michelson et al., 2020). Transdiagnostic approaches, germinating through 2004 and beyond, take into account social, psychological, neural and biological factors responsible for distress rather than a single disorder-based focus (Mansell and Tai, 2022). While targeted interventions can address adolescents at high risk, universal, transdiagnostic interventions in schools have proven to be crucial in promotion of well-being, aiming to prevent the onset of mental health issues among a larger young population (Wang et al., 2024). We do not, however, know much about which of these interventions would be most adapted to adolescents in underserved Indian community settings marked by dense populations, scarcity of resources and limited awareness.

SAMWAAD was an initiative woven into Society for Nutrition Education and Health Action’s (SNEHA) adolescent health programmes in Mumbai and Thane’s informal settlements. SNEHA’s Empowerment Health and Sexuality of Adolescents (EHSAS) programme works with adolescents aged 10–19, using a positive youth development approach (Ajgaonkar et al., 2022) to engage young people, parents, communities, stakeholders and public institutions (healthcare providers, police, educational institutions and civic representatives). The programme aims to enhance adolescent health (physical, sexual and mental) and promote gender equality. Activities include community mobilisation, mental health screening and referrals, catalysing youth facilitators, collective learning for adolescents on gender, sexual/reproductive health, citizenship, careers, health and nutrition, as well as strengthening public health systems. Operational in Dharavi, Kandivali and Kalwa, it serves more than 4,000 adolescents (2021 estimates: Kalwa – 1,500, Dharavi – 1,432 and Kandivali – 1,000) and 2,500 parents. The mental health intervention features preventive work such as sessions on emotional resilience, mental health awareness, referral to clinical psychologists and group sessions promoting help-seeking behaviour.

A situational analysis study, triggered by the challenges posed by the COVID-19 pandemic, informed the piloting of mental health screening tools to identify common mental disorders among adolescents. Assuming that a lack of awareness about adolescent mental health and limited community-level support acted as primary barriers, non-specialist workers (NSWs) were envisioned as pivotal in facilitating mental health discussions, providing psychoeducation, and ensuring early identification and referral. This led to an implementation research study to evaluate the feasibility and acceptability of a stepped care model, employing community-based NSWs, to enhance access to psychosocial treatment for adolescents in Mumbai and Thane’s informal settlements. Guided by questions on perceptions about mental health, screening tools and the development of a stepped care model, the study aimed to address issues, including social determinants of mental health and the role of NSWs in managing distress, identifying common mental disorders and making appropriate referrals. The iterative process involved understanding the perspectives of young individuals, families and NSWs. Considering literature that points at social, psychological and biological factors that underlie distress and mental health issues, *SAMWAAD* (dialogue/conversation in Hindi and an acronym for **Stepped cAre Model to Work on All that Ails the minD**) was a transdiagnostic stepped care model to deliver psychological treatment for adolescents through NSWs in urban vulnerable community settings.

## Methodology

The **underlying framework** for this study drew upon:

1. **The WHO Comprehensive Mental Health Action Plan 2013–2030 (2021),** emphasising non-specialised health workers for “comprehensive, integrated and responsive mental health and social care services”; it emphasised the acceptability and accessibility of community-based mental health resources as against healthcare facilities (Kohrt et al., 2018) and helped us map our study accordingly in identifying NSWs from urban vulnerable settlements
2. **The Friendship Bench** that trained and supervised lay health workers (LHWs) in Zimbabwe, providing psychosocial first aid to address common mental disorders in a community setting (Chibanda et al., 2015); with “no one to talk to openly” being a common refrain from our situational analyses with young people, “the friendship bench” model helped us build capacities of NSWs to listen to young people and assess their concerns while talking to them
3. **The *Atmiyata* model** piloting a community-based intervention with volunteers to address distress/common mental disorders among adults in rural India (Joag et al., 2020) encouraging us to engage NSWs to work with young people with no other such precedence for this population segment

**Figure 1.**
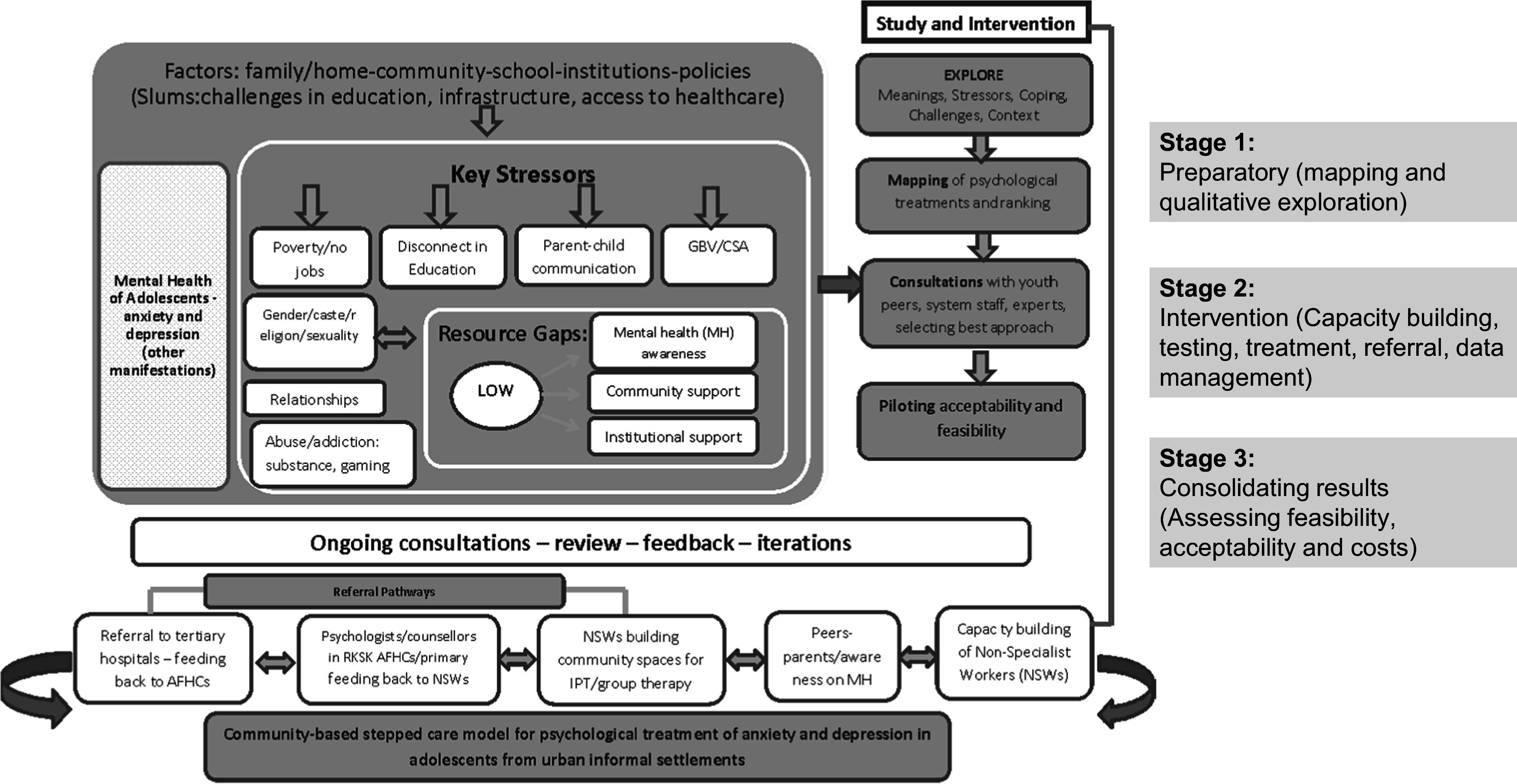
Conceptual framework for the SAMWAAD study.

The conceptual framework for the study drew upon the above literature and lived experiences of young people revealed in the situational analysis. The relationship between poverty, social identities and discrimination, inadequate educational, infrastructural and social protection support, gender-based violence, strained relationships with parents or with friends, intimate partners and substance abuse is depicted in Figure 1.

This study aimed at understanding the processes in implementing a stepped care model for common mental disorders in adolescents that include (a) a feasible screening approach by NSWs, (b) an adapted psychosocial treatment plan and (c) referral pathways; evaluate the feasibility and acceptability of this pilot intervention.

Since the study observed a cohort of young people and NSWs over a period of time, a prospective (Ranganathan and Aggarwal, 2018) forward-looking design bode well for the ensuing analyses and recommendations for future action. It warranted a combination of qualitative and quantitative paradigms to be considered. As befits a qualitative paradigm, the literature reviewed and the models from the WHO mental health action plan, friendship bench and *Atmiyata* provided sources for the conceptual framework. For the first objective, a qualitative paradigm that requires empirical data collected systematically from the field (Glaser and Strauss, 1967) helped us understand stressors, processes and discern certain patterns to add to theories on a stepped care model engaging NSWs to address adolescent mental health. Evaluating acceptability required discovering perceptions of young people, parents and NSWs through qualitative methods. Evaluating feasibility prompted quantitative inquiry aimed at enumerating the prevalence of common mental disorders, severity of conditions and the need for appropriate mental health intervention and referral based on screening results and scores secured by NSWs.

### Setting

**Figure 2.**
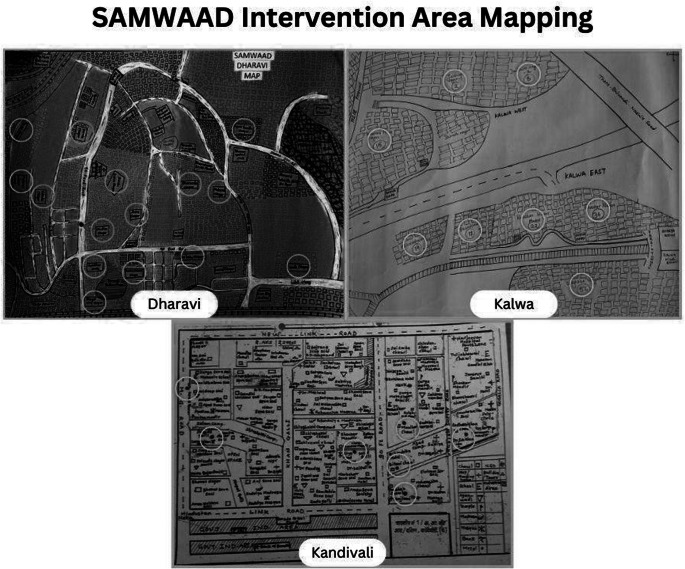
Maps of the three communities created by the implementation team.

The study was carried out in three informal vulnerable settlements of Maharashtra: Dharavi and Kandivali (Mumbai district) and Kalwa (Thane district). These settlements (refer to Figure 2 for hand drawn maps of the communities) are densely populated and have large migrant populations. Dharavi being the oldest settlement among the three belongs to families from different regions of India speaking their native tongues. However, a majority of the population uses functional Hindi for general communication. In both Dharavi and Kandivali, young people are most fluent in Hindi with a smattering of simple English terms thrown in; 90% of the population in Kalwa comes from native Hindi-speaking families – migrating from Uttar Pradesh.

Communities face challenges accessing basic health and education services (Adukia et al. 2022). In addition to inadequate infrastructure, adolescents face gender-based social constraints and violence at home and in public spaces. According to India’s last National Family Health Survey (NFHS 52019–20; International Institute of Population Sciences 2019–2020a), Thane district, in Maharashtra where the study’s Kalwa community is located, shows a sex ratio of 982 females per 1,000 males. Mumbai district (NFHS 52019–20; International Institute of Population Sciences 2019–2020b), where Dharavi and Kandivali are located, shows a sex ratio of 939 females per 1,000 males. While Thane shows 55% of women with 10 or more years of schooling, it is higher in Mumbai at 71%. In total, 55–58% of adolescent girls in both districts were found to be anaemic. Tobacco usage was found among 15% of young men in both districts, while 20–24% showed alcohol consumption. As compared with Mumbai with 50% households with no proper sanitation facility, Thane fared better with 30% households in this category. While both the districts had 96% to 100% households with improved drinking water sources, the population we worked with had 40% households (out of 1,600) in Kalwa and 20% (out of 1,500) in Mumbai with no access to drinking water on a daily basis.

### Recruitment

Five hundred young people and 500 parents – one for each adolescent – recruited from a larger pool of 4,000 programme participants, were included in the study. NSWs comprised community organisers at SNEHA – paid frontline workers – and unpaid community volunteers. All the NSWs came from economically marginalised backgrounds, and out of the 52 people, 40 were exposed to aspects of mental health concerns due to their association with SNEHA’s EHSAS programme. The remaining 12 had been associated as parents of young people or community volunteers and had not participated in sessions or workshops on emotional resilience or mental health and well-being, except for witnessing community campaigns on related topics. Piloting a stepped care model to assess feasibility and acceptability assumes a task-shifting approach with NSWs providing psychoeducation, screening, first-level counselling and referral services. The selection criteria included motivation to engage in social or community work, demonstrated association with SNEHA, comfort with writing, age, adaptability, interest in communication, openness to learning and sensitivity to others’ concerns. Additionally, candidates were required to be able to commit time for this work, with availability during weekends if currently employed, and to be geographically proximate to the adolescents served by the programme.

Each NSW was assigned 10 young people based on the population ratio. Functional Hindi was the language used by the NSWs to engage with adolescents and their parents. The Strengths and Difficulties Questionnaire (SDQ) mental health assessment tool, interview and Focused Group Discussion (FGD) guides, consent forms, facilitators’ manual and fidelity checklists for NSWs were translated from English to simple Hindi such that young people, their parents and NSWs could understand them easily. The implementing team, researchers, facilitators and clinical psychologists, all spoke simple Hindi and Marathi if required. Capacity-building sessions with NSWs, counselling sessions with young people and data collection exercises were carried out in the same languages. Simple English terms that young people and NSWs were familiar with were used if needed.

### Tool employed for assessment of mental health concerns

The SAMWAAD study employed the SDQ, a 25-item tool validated in the Indian adolescent context to assess behaviours, emotions and relationships of respondents (Goodman, 2001; Stevanovic et al., 2015), to understand five factors. *Emotional symptoms* point towards headaches, stomach aches, worries, fears, nervousness and sadness; *Conduct problems* highlight behavioural aspects such as temper tantrums or obedience, fighting or bullying, lying, cheating and/or stealing; *Hyperactivity inattention* points towards restlessness, fidgety behaviour, distraction against good attention span amidst tasks; *Peer problems* pick up playing alone, having just one friend or adult friends and being liked by others; *Pro-social behaviour* brings out a caring attitude, helping others, sharing and being kind to younger children, etc. (Hall et al., 2019). The SDQ when piloted with the sample population was found to reveal a range of factors affecting mental health rather than a specific disorder, thus remaining true to a transdiagnostic approach. NSWs found it easy to talk to young people about the five items to understand both internalising and externalising factors, thus enabling them to understand mental health contextually. A combined cut-off score of 17 and above for Emotional symptoms, Conduct problems, Hyperactivity inattention and Peer problems indicated concerns related to anxiety, depression or aggressive behaviour, prompting referral to a clinical psychologist. NSWs were also trained to report even if one or two factors showed a higher cut-off. A higher cut-off for Pro-social behaviour indicated fewer concerns and lower risks.

### Inclusion and exclusion criteria

All the adolescents in the age group of 11–17 years, enrolled with the EHSAS programme in Dharavi, Kandivali and Kalwa and with their parents consenting to and assenting to participate in this study, were included. For youth who were 18 years and above, all those who consented to participate in the study were included. Parents of young people enrolled with the EHSAS programme and consenting for their children to participate in the study were included. All the NSWs and clinical psychologists involved in the study and consenting to participate in providing information were included.

Criteria to exclude involved young people who did not give assent and parents of young people who did not consent to their child’s participation in this study. Adolescents with neurodevelopmental concerns and severe mental disorders as identified by clinical psychologists during previous interventions were not included in the study. Adolescents who screened positive for common mental disorders but did not wish to receive the psychological treatment from NSWs were excluded as well.

### Sampling

Five hundred young people (11–17 years) from among the EHSAS cohort of 4,000 adolescents who met the inclusion criteria were sampled through stratified random sampling based on their location (Dharavi, Kandivali and Kalwa) and their gender. Exclusion criteria were further applied on the sampled list to arrive at the final sample. Over-sampling was considered to ensure compensation for dropouts. All of them were residents of Dharavi, Kandivali and Kalwa, 51% of them being girls and 42% boys.

A sample size of 500 parents was considered for the qualitative aspects of the study. To engage them in in-depth interviews (IDIs), we resorted to purposive sampling and considered the results of the SDQ screening tests to include a representation of adolescents showing high risk, borderline risk or no risk. We also considered representation across parents of girls and boys and affiliated to different religions. A total of 52 NSWs comprising 20 frontline field workers, fifteen young people, ten parents and seven other frontline workers were included in the sample.

### Data collection

Quantitative data were gathered through the SDQ, pre–post-tests to assess retention of conceptual knowledge from the sessions with NSWs, fidelity rating ascribed to NSWs and monitoring indicators captured periodically. Quantitative data were gathered from all young people and NSWs. In total, 413 adolescents were screened using the SDQ. At the end of the intervention, 37 NSWs evaluated themselves on the self-rating fidelity tool and 29 NSWs were evaluated by clinical psychologists who employed the expert-rating fidelity scale.

Purposive sampling was employed for qualitative data collection ensuring maximum diversity across gender, age and contextually relevant criteria such as religion and school status (out of school/informal school). For post-intervention qualitative evidence, parents of adolescents falling in no risk, borderline risk or high risk were considered for IDIs. Data were captured through IDIs, FGDs and observations through three phases – (i) situational analysis (42 IDIs and 16 FGDs), (i) post-capacity-building sessions (5 IDIs with clinical psychologists and facilitators and 3 FGDs with NSWs), (iii) mid-intervention phase (16 IDIs and 13 FGDs) and (iv) post-intervention phase (23 IDIs and 15 FGDs), covering 191 young people, 110 parents, 20 doctors and frontline workers and 33 NSWs.

### Analyses

For quantitative data, all the study participants were identified with unique identification numbers/pseudonyms. The primary analysis was based on individual records. The data were described and summarised using mean and standard deviation. Non-normally distributed variables were reported using median and interquartile range. Categorical variables were reported using frequencies and percentages. Categories used for identifying relationships with primary outcomes included socioeconomic status and uptake of treatment support based on the referral pathway.

Each IDI and FGD was coded using the NVivo-10 qualitative analysis software to unearth themes. Data-driven codes emerging from observations were added to the above and then presented with priority accorded, based on frequency of occurrence through the evidence. In addition, summary of supervisory notes for each NSW was analysed to discern patterns and divergences regarding competencies and skills acquired.

NSWs took a self-reporting assessment (Likert scale) complemented by a fidelity checklist assessment to broadly understand competency levels and areas of growth at the end of the intervention. The Likert scale showed a mean score of 77 indicating the average self-assessed fidelity across all NSWs. Of them, 35% were in the lowest tertile indicating a perception of being less effective, 38% in the middle tertile indicating a moderate effective perception of themselves and lastly 27% in the highest tertile indicating a perception of being highly effective. The expert fidelity rating, assigned by clinical psychologists showed a mean score of 65 indicating moderate competency.

## Results

SAMWAAD was a pilot study with a multi-tiered referral system through which a) NSWs offered psychological first aid, b) clinical psychologists mentored NSWs and addressed their referrals, and c) psychiatric referrals for severe cases were made. The implementation involved a **one-year pilot** intervention spanning two phases, preceded by a formative qualitative study.

### Formative study: Understanding key stressors affecting mental health of young people

A qualitative situational analysis was undertaken in 2020–2021, to understand perceptions around mental health and the stressors faced by young people from urban vulnerable community settings. Programme monitoring data showed that most young people came from households with income not exceeding Rs. 15,000–Rs. 20,000 (USD 180–245) per month. The formative qualitative study found that poverty and structural inequality were prominent across all three settings with families caught up in debt traps and out-of-pocket expenses for health. Young people here were mostly first-generation learners. Fathers worked in the informal sector as carpenters, drivers, daily wage labourers, tailors, etc. Most mothers supplemented household incomes by working as domestic helpers, cooks, tailors or by taking up odd assignments at home like stitching or garland making. Most people in the settlements came from a mix of Muslim, Dalit and other backward caste demographics.

**Figure 3.**
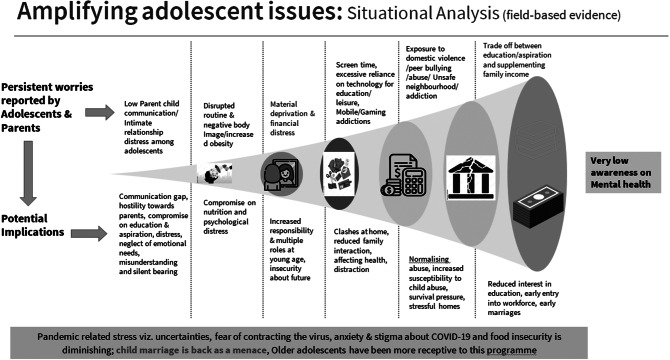
Findings from the formative study.

Terms such as “*paagalpan*” (madness), “depression,” “tension” and “*naatak*” (drama) were commonly used in a derogatory manner, trivialising the significance of mental health. Most young people felt that mental illness was something that “cannot be seen or understood.” They were not aware of any “doctor capable of treating such cases; the only way was to take such abnormally behaving people to a Baba (traditional healer), a *dargah* (shrine/mausoleum of Muslim saint with perceived magical powers) or to others who could conduct *jhaad phunk* (exorcism).” Some of them associated mental illness with “captivity in a ‘mental hospital’” to keep their “behaviour under control and avoid harming others.” The findings from this phase have been summarised in Figure 3.

Based on the qualitative findings, two key aspects emerged:

#### Cultural vulnerabilities resulting in high conformity – Low communication orientation in families

Most families in urban informal settlements came across as protective, practised limited communication beyond the routine and expected social conformity (Koerner and Fitzpatrick 2006), especially from young people, girls and women. Respect for, fear of and obedience to “authority” – especially towards parents – were of prime importance. Parents expected the same from their children because they grew up with “fear that was used to hold regard to” or “a lot of inhibitions towards and respect” for their “elders.” The confusion of “feeling shame at one’s undesirable actions” and “respect for elders” led to accepted expressions of shame for one’s actions that spread across households and neighbourhoods. Insufficient communication between parents and young people emerged as a pressing issue, underscoring the imperative for intervention by NSWs. The fabric of familial connections, crucial for the holistic development of adolescents, seemed to be lacking due to various factors such as survival pressures, busy schedules and inadequate understanding of effective communication. “*As a parent, I am not able to identify or understand when the child feels disturbed or what goes on in their mind.*” (mother, Kalwa).

Young people “without shame” were perceived to have “loose morals,” were “modern” and “free.” Culture expected a “good child” to comply and feared adolescents following the “*galat raasta* (wrong path).” Youth who “fell in love,” “answered back” at home, “disobeyed the elders” or “roamed around” would have taken the “wrong and prohibited path.” Some mothers feared about “girlfriend and boyfriends at the age of 12–13; they were looking at the internet and doing so.” Parents said that they “are afraid of friendships as they could be a bad influence” (mother Dharavi). Friendship with the opposite sex or meeting them alone was not allowed, and conformity was measured in terms of young people who “focused on their studies, did not go around much in the community” or did not express emotions of anger or irritation (“*chid-chid*”). These rules were imposed differently across genders.

*My daughter also doesn’t go outside, she remains at home, she might argue with me, I will scold and shout but nothing else, she will go to school and then return home, the girls of her age come but she doesn’t go with them; my daughter knows her boundaries (mother, Kalwa).*

Other conformity norms emerged especially for adolescent girls (Daruwalla et al. 2018). There was high emphasis on “honour” of the family that correlated with behaviour of adolescent girls, often resulting in trading off agency in the form of (i) restriction on aspirations, (ii) restrictions on expression, recreation, mobility and play and (iii) pressure to perform gender-based roles.

*In my conservative family, girls are not encouraged to study. I envision myself becoming a teacher because I am passionate about education and want to make a positive impact on the lives of others. Teaching provides an opportunity to inspire and empower future generations. However, my father believes that girls should not go out and study, which creates a conflict within the family (adolescent girl, Dharavi).*

#### Migration, poverty and gender roles

Cultural vulnerabilities were exacerbated by the pressures of household chores on girls. As both her parents worked, a 13-year-old Kalwa girl stated that her major stressor was “*cooking at home; my 15-year-old brother does not help; he changes his clothes five times a day, I have to wash all of them; I’m responsible for running the entire household.*” Field evidence revealed that parents did not explain their decisions to youngsters. There was no effective conflict resolution since conflicts were perceived negatively. Impoverished migrant families in urban informal settlements conformed because of material and cultural vulnerability. Control was enforced by immediate family members, extended family, neighbours and those in distant villages. Women – mothers of young people – had been staying in Mumbai for 15 years and had not “gone beyond Dharavi” even for a city tour or pleasure trip. Their sole purpose in life was to “ensure happiness for their husbands, secure education for their children, save money from whatever their husbands earned and keep the home and the hearth going.”

Many young people internalised the pressures by excelling in school and building a career, while others worked while learning. For a few others, it meant “giving up.” They expressed their tensions through “talking openly” with peers or sharing their worries with their mothers, siblings and cousins. However, some coped by “not talking to anyone,” sobbing or “going out when angry.” Some also resorted to “breaking things” and “hitting my sister.” While smartphones provided entertainment and coping, they also contributed to further isolation and alienation among young people.

### Processes involved in implementing the SAMWAAD stepped care model

Informed by the formative study, NSWs carried out the intervention based on availability, basic literacy and motivation to address mental health concerns. An intervention manual was designed based on WHO’s problem management plus manual (Dawson et al., 2015; Problem Management Plus (PM+), 2018), a cognitive behaviour therapy (CBT)-based problem-solving counselling process (Oud et al. 2019). To prepare the NSWs, the research team, comprising ethnographers, with training in instructional design to develop educational content, social workers and clinical psychologists, executed this manual to build the capacities of NSWs. They employed Beattie’s model (Beattie, 1995) on creating an environment promoting reflective learning, critical thinking, creativity, interpersonal relationships and attitude change through process learning. Capacity building of NSWs spread across eight sessions and workshops of two hours and full-day slots. Designed as a series of Training of Trainer (ToT) sessions and workshops, topics included building safe spaces, understanding emotions and empathy, introduction to mental health, mental disorders among adolescents, role of NSWs, listening and paraphrasing while counselling and using basic screening tools. Picking up clues on material stressors to address social and structural determinants of mental health was also discussed, as were referral systems and networks that could be accessed by young people. For over two months, this capacity-building series was spread across Saturdays through sessions or half-day long or full-day-long workshops. The NSWs received refresher module content via WhatsApp, for ease of use, based on their convenience and also participated in three refresher sessions during the intervention.

NSWs carried out the intervention with young people from informal settlements in Dharavi, Kandivali and Kalwa. This included mental health screening using the SDQ. If the screening process found young people to be at “high risk” in any one of the SDQ sub-scales, or on the overall score, they were referred to the site-wise clinical psychologists. Cases that required further referral for strategic management were referred to psychiatrists. The remaining young people were provided with psychosocial first aid by the NSWs. During this phase, the NSWs were supported by (i) an interdisciplinary supervisory committee to help address social determinants such as the need for educational sponsorships and eligibility documents to apply for public welfare schemes and (ii) site-wise clinical mentoring support. This supervisory committee included the principal investigator, qualitative researchers, senior members of the EHSAS implementation team and three clinical psychologists. Across 10 months, the supervisory committee met on 15 occasions, besides three site-based meetings. Site-wise clinical mentoring support was provided by clinical psychologists. It included observations of NSWs conducting sessions and a monthly meeting to review cases and respective management of the same. Twenty-two observation sessions were conducted, and every NSW participated in three to four supervision meetings on an average.

#### Assessment of young people’s mental health and intervention by NSWs

Although the intervention was envisioned to begin with 500 adolescents, 413 adolescents were screened across three locations. The reasons for dropout and the site-wise number of adolescents enlisted are as shown in Table 1.

**Table 1.** Cumulative count of adolescents enlisted and screened (using the SDQ tool) by NSWs

Area	No. of adolescents identified	No. of adolescents enlisted and screened by NSWs	No. of adolescents received support from NSWs	No. of adolescents referred to CPs
Dharavi	200	170	91	35
Kalwa	200	166	93	49
Kandivali	100	77	43	15
**Total**	**500**	**413**	**227**	**99**

*Note:* Reasons for drop in numbers: Migration: 35% Non-consent: 28% Time constraint of NSWs: 20% Time constraint of adolescents: 17%.

**Figure 4.**
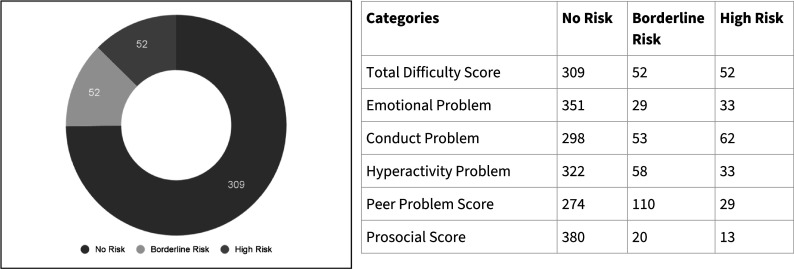
Prevalence distribution of high risk, borderline risk and no risk based on the SDQ screening by NSWs, based on “total difficulties score” and “sub-scales” (N-413).

Of the 413 enlisted adolescents, the “total difficulties score” from the SDQ showed that 52 (12.5%) adolescents were at “high risk” of mental disorders, 52 (12.5%) were categorised as “borderline risk” and 309 (75%) adolescents were not at risk and needed distress management, as highlighted in Figure 4.

Prevalence of mental health concerns could be understood from the total difficulty score, though intervention with adolescents was planned based on sub-scale score and sociocultural context. “Conduct” problems were present in 15% of the adolescents, and borderline peer problems were seen in 27% of adolescents. Considering the subscale score and history including abuse, grief and suicidal thoughts, those in need of intensive psychotherapy were referred to clinical psychologists. Cases that required further referral for strategic management were referred to psychiatrists.

NSWs counselled adolescents showing “no risk” and “borderline risk” on the SDQ. Two hundred twenty-seven adolescents were counselled by NSWs, 99 adolescents were referred to clinical psychologists and one referral was made to a psychiatrist. The core psychological intervention provided by the NSWs was guided and supported by the framework from WHO’s PM Plus manual (2018) integrated with CBT principles (WHO’s PM Plus manual 2018). The intervention involved a problem-solving approach along with behavioural management. The core psychological interventions were carried out within a phase-based framework with rigorous supervision processes and handholding from clinical psychologists trained in cognitive behavioural interventions.

In the first phase, NSWs interacted with the adolescents and their family members, explained the study intervention, built rapport, understood the present psychosocial context, and experiences of adolescents, and administered the SDQ. Lastly, NSWs discussed about each adolescent with clinical psychologists and outlined steps of action and support for the next phase of intervention. During the assessment and discussion, if the adolescents were found to be at high risk including the presence of abuse, violence and suicidal thoughts, they were directly referred to the clinical psychologists for psychotherapy and clinical support as required.

In the second phase, NSWs strengthened rapport and identification of problems and stressors. Once identified, they met clinical psychologists to discuss steps for managing stress and problems identified in keeping with the framework of the WHO’s PM Plus manual. This was supported with systematic supervision and one-to-one training with NSWs on problem-solving (listing problems, defining, brainstorming helpful strategies, devising action plans and revising), and managing stress (slow breathing and grounding exercises). On the need of adolescents’ problems, behavioural techniques such as contingency management and token economy were used.

The second phase included psychoeducation such as building a safe space for conversations and discussing the identified emotional and behavioural concerns in the adolescent’s respective psychosocial context. Building parental awareness of the concerns identified and discussing their questions and curiosities included educating about coping skills, readjustment of expectations and communication patterns and supporting the family with psycho-social support. Lastly, the causes of problems identified and management steps were discussed with parents.

In the third phase, after the completion of problem-solving steps and managing stress, a debriefing and introduction of termination were carried out under the supervision of the clinical psychologists. The process of termination was supported by a review of resources and learnings built and learned. The SDQ was administered again at the end to track improvement. Lastly, closure was informed and discussed with parents or caregivers.

#### Acceptability: Shifts in young people’s understanding of mental health and help-seeking behaviour

Data from the SDQ administered after the intervention among 175 adolescents showed that a large proportion of the adolescents (20%) moved from “borderline risk” to “no risk.” This change was seen across all three locations, with Kandivali seeing the highest proportion of change. However, the proportion of adolescents at “high risk” remained the same as highlighted in Table 2.

**Table 2.** Pre- and post-intervention data from the SDQ

Categories	Dharavi				Kandivali				Kalwa				Total			
	Pre-SDQ		Post-SDQ		Pre-SDQ		Post-SDQ		Pre-SDQ		Post-SDQ		Pre-SDQ		Post-SDQ	
	n	%	n	%	n	%	N	%	n	%	n	%	n	%	n	%
High risk	3	4%	3	4%	0	0%	1	3%	3	4%	2	3%	6	3%	6	3%
Borderline risk	24	33%	12	16%	16	48%	3	9%	21	30%	11	16%	61	35%	26	15%
No risk	46	63%	58	79%	17	52%	29	88%	45	65%	56	81%	108	62%	143	82%
**Total**	73	100%	73	100%	33	100%	33	100%	69	100%	69	100%	175	100%	175	100%

Largely, the SAMWAAD intervention opened up spaces of communication for young people, while drawing support from their parents, and it established the relevance of (i) acceptance of the need to address emotional and mental health concerns, (ii) the importance of listening to young people and (iii) the recognition of help-seeking behaviour among young people.

There was an evident increase in understanding and articulation of mental health among young people and their parents. Post-intervention, most young people addressed mental health using words such as “problem,” “tension” and “stress” and no longer alluded to “*naatak* (drama),” “*buri nazar* (evil eye)” or “possessed,” as evidenced in formative situational analysis. One young person specifically said “*it is the health of the mind*” and another said “*brain is under pressure.*” Across all interviews, an acceptability of speaking openly about these concerns was revealed.

*“Someone was taking interest in me, and wanted to know what I think and feel; I had never realised that these can be important for others*,*”* emerged as a common theme across conversations. Interactions between young people and NSWs covered a spectrum of concerns they had – academic pressure and anxieties, career aspirations, conflicts with friends, feelings of suffocation, irritation, anger and alcoholism at home. With some, deeper conversations on grief and abandonment took place. Parents noticed an increase in agency among young people, and their ability to draw boundaries. One young person particularly stood up against violence at home “*when his father abused him, and asserted that his father must not abuse him verbally while talking to him; he had also started standing up for me, telling him that I will dial 100 and call the police if you abuse her*” (mother).

Regular and deeper than usual conversations with NSWs were resulting in noticeable behaviour change as parents found young people to be “*politer and calmer.*”

*after I insisted that Tai (sister-NSW) talks to him at least once to make him understand the importance of studying rather than taking up a job; She must have been able to convince him, since now he is back to concentrating on his grade 12 studies (mother, Dharavi).*

It was insightful to note how parents’ trust towards SNEHA “*for being with us throughout our troubles*” allowed them to “*leave their children completely in the safe hands of the didi and Sir (NSWs).*” This trust, notably strong, played a pivotal role in fostering greater receptivity to the intervention by NSWs in the stigmatised realm of mental health.

The results from the effects of the Friendship Bench (Chibanda et al. 2016) intervention in Zimbabwe demonstrated the trust reposed in the contextually relevant care of health workers. Likewise, for the SAMWAAD study, beyond assisting young people through their concerns and anxieties, **NSWs also seemed to have become influencers for parents** who mentioned that they were able to understand their children better and had started reflecting “*on my own actions; Didi helps us understand how we should be behaving before our children.*” Most parents felt that while the cultural context did not allow their children to open up to their parents, “*our children felt safe that they had someone they could go to, someone they trusted and was older and could guide them; the didi/sir talk to children as friends and children pour out their hearts freely.*” This perceived safe space for open dialogue, and emotional expression significantly contributed to the overall acceptability of NSWs. Both adolescents and parents agreed with the need for and the benefits of “*talking about feelings and emotional wellbeing*” as taken up by NSWs during the SAMWAAD intervention.

### Situating NSWs: Adaptation of psychosocial treatment for adolescents

#### Personal and professional growth of NSWs

Out of the total 52 NSWs enabled to work with young people, 48 were retained for the intervention process based on availability and interest. In context of the urban informal settlements, accepting that the children in the community knew and recognised the NSWs more led to a stronger rapport between them, since the NSWs were also engaged in facilitating other “*activities with the young people; I visit their homes for nutrition counselling and also know their parents; it is due to this familiarity that they are not hesitant to talk to me*” (NSW, Kalwa). NSWs expressed a deeper understanding of the community’s context, cultural nuances and individual circumstances, enabling them to connect with the children better. This nuanced understanding stemmed from the fact that the NSWs were from the same communities and contexts.

However, in spite of NSWs belonging to the same communities, parents were often suspicious owing to the stigma and ignorance surrounding mental health; parental curiosity and a lack of communal privacy made it difficult for NSWs to maintain confidentiality. Some parents were hesitant to sign consent forms, expressing a deep-rooted fear of authority figures. Vulnerable and impoverished migrant families were anxious about potential future obligations, alienation in “*pardes* – foreign land” or negative consequences “*since we don’t know how our signatures might be used.*” A few were not comfortable with male NSWs holding “private conversations” with their daughters. Sessions with NSWs on respecting privacy and confidentiality enabled them to explain the study to parents of young people over a few meetings and gain trust. It was easier for SNEHA staff NSWs to do this owing to their existing relationships in the communities. Unpaid NSWs took time but eventually were welcomed by parents and young people alike.

Even though literature supports the benefits and need of task shifting within the broader public health system and mental health professionals, a distinct insistence on maintaining separate professional identities was experienced throughout the study. Some specialised mental health professionals seemed “*worried about this; I noticed that even though the intention/motivation is great, practically they seem nervous in the field.”* While some clinical psychologists were apprehensive about the idea of a task-sharing approach, a few others gradually shed their “biomedical” lens and challenged their own ideas, as they started valuing the contribution of the NSWs. The camaraderie between clinical psychologists and NSWs was evident with them leaning on each other since

*the engagement of NSWs has helped us increase the coverage of young people with distress and I must admit that most of them have overcome their own stigma attached with mental health and are assisting adolescents and their parents in addressing mental health concerns; we get more referrals nowadays* (clinical psychologist, EHSAS Kandivali centre).

The NSWs expressed how their perceptions about mental health had gone through a sea change. They were “*able to draw boundaries in their personal lives, and were learning to say ‘no’*,” “*I use more eye contact during conversations and feel more confident in holding discussions*” (community volunteer). The result of reduced stigma showed a drastic change in self-awareness, self-care and interpersonal relationships within families of NSWs. While some spoke about improvement in the ways in which they “now” interacted with their children and husbands, others reported increased confidence, improved listening skills and the ability to identify and mediate interpersonal issues.

After completing the capacity-building sessions and the intervention process, community organisers employed with SNEHA, doubling up as NSWs, mentioned that,

*“the skills learnt through SAMWAAD have helped us connect better with young people across the cohort - it is strange that we have been working with them for so long and yet, we seem to understand them better only after becoming barefoot counsellors (BFC)” (NSWs); “I now understand separate aspects of the same person whom I have known since earlier; “actually, we were simply seeing signs before, but they were being ignored because our focus was limited to our work. Now, through BFC, I have learned about all these things; “even before becoming a barefoot counsellor, I used to talk to children, but now I know how to communicate step by step, what to ask, and even our way of speaking has changed. Children are enjoying the group sessions better now.” (NSWs from across Dharavi, Kandivali and Kalwa).*

The NSWs showed proficiency in identifying subtle cues and providing assistance to individuals, even to those not enrolled in the programme.

*because of my learning as a BFC, I can help people; There is a kid from 10th standard who comes for my tuition classes; she doesn’t have a mother, it is some personal matter. She faces a lot of pressure from her grandmother. I understood a little bit from my friends and after talking to her, I understood better. Without asking Ma’am, I used the SDQ tool with this girl during a conversation; she showed high risk in all the parameters and I referred her to the clinical psychologist (21*-*yr-old NSW, Kalwa).*

The WHO Mental Health Action Plan 2013–2030 (2021) advocates the promotion of care through recovery-oriented community-based mental health and social support services. In the SAMWAAD pilot intervention, we found that the NSWs effectively filled the gap of the scarcity of trained human resources. They learnt with enthusiasm, challenged their personal stigma, assisted young people and their families and made appropriate referrals to clinical psychologists. During their engagement as NSWs, mobile phones were provided to facilitate communication with adolescents and the supervisory team. In the absence of monetary compensation, these phones with a nominal recharge paid by SNEHA were an incentive for the NSWs.

## Discussion

The SAMWAAD implementation research aimed at understanding if a stepped care model on adolescent mental health involving NSWs would be acceptable and feasible. Based on the findings, we saw that “Acceptability,” a critical aspect of access, was closely tied to patient–provider trust (Dyer et al., 2016). The social and cultural distance between healthcare systems and users influences this dimension. SAMWAAD’s acceptability criteria hinged on SNEHA’s reputation and standing in the community, playing a critical role in fostering trust between community members and NSWs. Speaking to the positive results of contextually relevant caregivers cited in the *Atmiyata* model (2020) and the Friendship Bench intervention (Chibanda et al. 2016), this pilot study carried out in urban Maharashtra echoed the relevance of lay counsellors assisting young people.

*Whatever you (SNEHA) do for our children would be for their good; you are always there for our children, why would we not come if you call us for meetings to explain or share information?* (Parent, Kalwa).

Respect for the cultural context, especially gender considerations, was imperative for acceptability. While the SAMWAAD study gently emphasised building agency of young people to seek help, it did not challenge the adherence to cultural norms. Instead, the intervention phase included working with parents to help them understand their children better. Importance was accorded to usage of simple and contextual language, acknowledging the impact of terminology on engagement. Akin to the *Atmiyata (*Joag et al., 2020) intervention where the “champions” conducted 4–6 low-intensity psychosocial sessions, the NSWs conducted at least 4 sessions with young people and their parents to identify distress and common mental disorders.

*When I discussed “MANASIK SWASTHYA” (mental well-being) with the families of adolescents, they responded positively. However, when I used the term “‘mental’ health,” the family members’ reactions turned negative, often associating stereotypes with the term* (CO NSW from Dharavi).

Feasibility considerations (Bowen et al., 2009) encompass time commitment, incentivisation, ease of documentation, adequate privacy and space, and community-wide mental health campaigns. The SAMWAAD study revealed a mixed review on aspects of feasibility. While NSWs were completely dedicated to learning and assisting young people and their families through distress, time commitment emerged as a primary barrier, affecting non-staff NSWs not on SNEHA’s payroll. Their economic considerations and family commitments, “*academic pressures at college level*” and “time required to look after my elderly mother in-law and household chores,” competed for the limited time they could allocate to the intervention.

The findings echo the emphasis of the WHO Mental Health Action Plan (2021) on structural determinants of mental health and mental disorders including social, cultural, economic (Lund et al. 2018), political and environmental factors such as national policies, social protection, poverty, discrimination, living standards, working conditions and community social supports. The situational analysis enabled the research and intervention team to build capacities of NSWs to understand stressors that existed in the real world, along with neurological and biological factors affecting mental health.

Evidence suggested a need for incentivisation of some NSWs. Almost all the NSWs were women, and they expressed that their families questioned the value of their work since “*You don’t even get paid for it.*” The NSWs acknowledged the need to “*explain and justify our work to our families*,” and with no monetary compensation, voluntary counselling became a practical constraint for them. SAMWAAD’s success in retaining motivated NSWs is attributed to tangible advantages provided, such as mobile phones, access to educational films and training certificates.

Implementation protocols must prioritise ease of documentation to enhance operational efficiency. It is recommended to adopt streamlined documentation protocols and implement targeted training programmes. Inadequate privacy and space present challenges in community-based stepped care frameworks. Identifying alternative secure spaces within the community is crucial for ensuring effective engagement while preserving privacy. The execution of community-wide mental health campaigns alongside primary initiatives increased visibility, creating a synergistic effect that reinforced the dissemination of vital mental health information.

Effectiveness was evaluated through experiential capacity building, supervision, observation, mentoring and addressing social determinants. Experiential capacity building focused on interactive sessions, role plays and learning through real-life scenarios. According to a facilitator, NSWs thrived on experiential learning, finding it to be the most engaging mode of learning during sessions. SAMWAAD’s success lay in engaging participants on a personal level, leveraging their experiences to create a profound and lasting effect. Visual representation, such as colour coding and visualising mental health on a spectrum, enhanced understanding and retention.

“*I will never forget the way the doctor explained mental health concerns in that video; I understand that mental stress is like a sprain (moch) in the foot where it aches, but you can still walk … you do need to seek help for the ache just like you must seek psychological help. Mental disorder is when something has broken, like a fracture, when you must be treated with medicines because your daily routine and life functions get disrupted due to the mental disorder*” (youth volunteer NSW). Participants appreciated the interactive nature of these sessions, as they provided tangible tools for effective communication and support. *“Experiences of meditation, stories, singing and drawing during sessions were interesting.”*

Supervision, observation and mentoring emerged to be crucial for improvement in the skills of NSWs. The process notes from different locations highlighted common themes and unique challenges, emphasising the significance of standardised approaches and principles. NSWs initially doubted their performance, especially with documentation emerging as a key limitation. With mentoring support, they became more confident and comfortable, and clinical psychologists and volunteers assisted them with written articulation of cases. The findings emphasised the need to address societal and systemic variables to support NSWs and improve mental health services for young people in their community settings. According to clinical psychologists, NSWs’ engagement with young people resulted in early identification, referral for moderate/severe cases and reaching a larger audience, including parents, with psychoeducation.

Qualitative analysis of fidelity ratings by supervisors found that NSWs were curious to learn and understand processes, had an in-depth understanding of consent and confidentiality as crucial aspects of mental health intervention, enabled safe spaces for adolescents, efficiency with SDQ administration and identification and made appropriate referrals with respective social stressors and determinants. Also, there was an increase in their sensitivity and alertness to mental health and related social determinants. Addressing social determinants involved proactive measures to prevent the onset of mental health issues among the less privileged population. SAMWAAD’s positive impact on NSWs’ capabilities underscored its potential in promoting better engagement and mental health outcomes for young people. A wide gamut of social protection referrals arose from the intervention, such as referral for night schools for out-of-school adolescents, sponsorship support for school and higher education and collaborating with SNEHA’s programme on Prevention of Violence against Women and Children (PVWC) to prevent child marriage.

The findings from the present study reflect some recommendations for further research and implementation for community mental health intervention. First and foremost, it is essential to hold a biopsychosocial conceptualisation for mental health and related concerns. It is essential to identify and contact adolescents’ and parents’ social determinants and cultural vulnerabilities as part of a mental health intervention. Second, the study reflects the need for a preventive approach for building a community mental health intervention. This will help to ensure psychosocial support to the adolescents and parents not in the high-risk or borderline mental health difficulties categories but are surrounded by stressors owing to their vulnerable contexts. Group psychotherapeutic initiatives have been planned in the next phase, along with a module on parent–child communication and normalising the experiences of parenting and building a safe and cathartic environment for parents. Lastly, there is an inherent need for awareness of preventive stepped care models among mental health professionals to influence public health communities. This will be taken up through AFHCs at the primary healthcare level and with teachers in school settings.

All the above aspects readily emphasise the role of NSWs who are based in community settings, can be approached by young people and their families easily and can become the conduit between psychosocial first aid and appropriate referral to expert mental health resources that may be distant to urban informal communities. The WHO Mental Health Action Plan 2013–2030 (2021) stresses upon Lay Health Workers (LHWs) as a potent workforce in strengthening community-based support systems as evident in the SAMWAAD study. With their own life examples wherein NSWs have been able to shed stigma around mental health, they are visible champions who can help community members hold ordinary conversations on mental health concerns and thus seek help early on. These findings are potentially useful in implementing a peer-worker-led model within the *Rashtriya Kishor Swasthya Karyakram.* The *Atmiyata* model (2020) anticipates that community volunteers would expect remuneration with the model scaling up in future. Considering the urban context, living costs of Mumbai, limited time constraints in daily living and varied motivation of NSWs, the SAMWAAD study also reflects the need for incentivisation beyond just knowledge and certification.

## Conclusion

It must be reckoned that a stepped care model involving NSWs is not essentially about task replacement but about task sharing. NSWs undertook psychoeducation as a significant preventive strategy, while identifying mental health stressors early on and making appropriate and timely referrals to professionals. Regarding adolescent mental health, the findings reveal the significance of (a) identifying and addressing social determinants that impact mental health, (b) working with families and communities to enable healthy conversations on adolescent mental health and (c) recognising the role of NSWs as intrinsic to community-based mental health interventions leading to appropriate referral. We recognise that this study carried out as a pilot intervention with a small sample of young people and with NSWs who largely comprised frontline workers at SNEHA had a limited scope. Scaling this model would require carefully planned protocols drawing upon the learning from this study.

## Data Availability

As per the data sharing policies of our organisation SNEHA, programme datasets can be shared online only after three years of completion of the programme or project. The Chief Executive Officer, Ms. Vanessa D’Souza (vanessa@snehamumbai.org) will be a non-author point of contact for data access.
