# Supplementary material for: “Helping myself empowered me to help young people better”: A stepped care model, with non-specialist workers (NSWs) addressing mental health of young people in urban vulnerable communities across the Mumbai metropolitan region in India
Source: Glob Ment Health (Camb). 2024 Oct 24;11:e95. doi: 10.1017/gmh.2024.96 (PMC11504945; doi:10.1017/gmh.2024.96)
Supplement: Peer review history

# Editor report: Recommendation: *“Helping myself empowered me to help young people better*”*:* A stepped care model, with non-specialist workers (NSWs) addressing mental health of young people in urban vulnerable communities across the Mumbai metropolitan region in India — R0/PR2

**DOI:** 10.1017/gmh.2024.96.pr2

**Affiliations:** Faculty of Business, Technology, and Education , Ilia State University, Georgia

No accompanying comment.

# Editor report: Decision: *“Helping myself empowered me to help young people better*”*:* A stepped care model, with non-specialist workers (NSWs) addressing mental health of young people in urban vulnerable communities across the Mumbai metropolitan region in India — R0/PR3

**DOI:** 10.1017/gmh.2024.96.pr3

**Affiliations:** Johns Hopkins University Bloomberg School of Public Health, United States

No accompanying comment.

# Editor report: Recommendation: *“Helping myself empowered me to help young people better*”*:* A stepped care model, with non-specialist workers (NSWs) addressing mental health of young people in urban vulnerable communities across the Mumbai metropolitan region in India — R1/PR5

**DOI:** 10.1017/gmh.2024.96.pr5

**Affiliations:** Faculty of Business, Technology, and Education , Ilia State University, Georgia

No accompanying comment.

# Editor report: Decision: *“Helping myself empowered me to help young people better*”*:* A stepped care model, with non-specialist workers (NSWs) addressing mental health of young people in urban vulnerable communities across the Mumbai metropolitan region in India — R1/PR6

**DOI:** 10.1017/gmh.2024.96.pr6

**Affiliations:** Johns Hopkins University Bloomberg School of Public Health, United States

No accompanying comment.
